# The Tri-phasic Role of Hydrogen Peroxide in Blood-Brain Barrier Endothelial cells

**DOI:** 10.1038/s41598-018-36769-3

**Published:** 2019-01-15

**Authors:** Chinchusha Anasooya Shaji, Bobby D. Robinson, Antonia Yeager, Madhava R. Beeram, Matthew L. Davis, Claire L. Isbell, Jason H. Huang, Binu Tharakan

**Affiliations:** 1 Department of Surgery, Texas A&M University Health Science Center College of Medicine and Baylor Scott & White Health, Temple, Texas USA; 2 Department of Pediatrics, Texas A&M University Health Science Center College of Medicine and Baylor Scott & White Health, Temple, Texas USA; 3 Department of Neurosurgery, Texas A&M University Health Science Center College of Medicine and Baylor Scott & White Health, Temple, Texas USA

## Abstract

Hydrogen peroxide (H_2_O_2_) plays an important role physiologically as the second messenger and pathologically as an inducer of oxidative stress in injury, ischemia and other conditions. However, it is unclear how H_2_O_2_ influences various cellular functions in health and disease differentially, particularly in the blood-brain barrier (BBB). We hypothesized that the change in cellular concentrations of H_2_O_2_ is a major contributor in regulation of angiogenesis, barrier integrity/permeability and cell death/apoptosis in BBB endothelial cells. Rat brain microvascular endothelial cells were exposed to various concentrations of H_2_O_2_ (1 nM to 25 mM). BBB tight junction protein (zonula ocludens-1; ZO-1) localization and expression, cytoskeletal organization, monolayer permeability, angiogenesis, cell viability and apoptosis were evaluated. H_2_O_2_ at low concentrations (0.001 μM to 1 μM) increased endothelial cell tube formation indicating enhanced angiogenesis. H_2_O_2_ at 100 μM and above induced monolayer hyperpermeability significantly (p < 0.05). H_2_O_2_ at 10 mM and above decreased cell viability and induced apoptosis (p < 0.05). There was a decrease of ZO-1 tight junction localization with 100 μm H_2_O_2_, but had no effect on protein expression. Cytoskeletal disorganizations were observed starting at 1 μm. In conclusion H_2_O_2_ influences angiogenesis, permeability, and cell death/apoptosis in a tri-phasic and concentration-dependent manner in microvascular endothelial cells of the blood-brain barrier.

## Introduction

Reactive oxygen species (ROS) is a critical regulator of multiple body functions in human health and disease. Traumatic or ischemic injury to the brain leads to the formation of excessive (ROS) and results in oxidative stress^1–3^. This can lead to further damage to the blood-brain barrier (BBB), the primary protective barrier of the brain leading to microvascular hyperpermeability and vasogenic edema followed by several adverse consequences^2–4^. Hydrogen peroxide (H_2_O_2_) is an important endogenous ROS implicated in health and disease but its role in the BBB is not clearly known. As in the case of ROS in general, H_2_O_2_ can be beneficial or detrimental to the body but the cellular mechanisms that lead to this beneficial or adverse effects are not clearly known and are highly controversial. H_2_O_2_ has been implicated in a variety of essential cellular functions such as cell/tissue regeneration, growth, proliferation, and cell migration, while its adverse effects include damage to proteins, lipids and nucleic acid and leading to cell death^5–7^. Considering this differential and controversial nature of the effects of H_2_O_2_, and the limited information available in its effect on the BBB, we have conducted a systematic study to analyze the various effects of H_2_O_2_ in the BBB endothelial cells, the primary components of the blood brain barrier. The significance of the BBB in regulating a wide variety of human diseases including traumatic and ischemic injuries and the roles played by oxidative stress and associated signaling pathways in the pathophysiology has been the subject of active research in the recent time^3,8,9^. The BBB consists of interendothelial junctions and specialized transporter systems that protect the brain and maintain homeostasis. These functions are achieved through three different junctions (adherens, tight and possible gap junctions)^10–12^. Tight junctions consist of multiple types of proteins such as occludin, claudins, intracellular zonula occludens-1 (ZO-1) and junction adhesion molecules. These proteins are important in maintaining the integrity of the barrier^10,13–16^. Recent studies from our laboratory has shown tight junction proteins particularly ZO-1 playing a major role in maintaining barrier integrity and permeability following traumatic brain injury and tight junction disruption is critical to BBB breakdown and hyperpermeability^8,13,17^ Furthermore, studies from our lab as well as by others have shown oxidative stress by ROS is critical to endothelial cell barrier dysfunctions^8,17,18^.

Reactive oxygen species have physiologic function and are known to be important in the regulation of angiogenesis, vessel growth from preexisting vessels. Previous studies show that H_2_O_2_ induces angiogenesis^19–23^. This occurs normally during embryonic development and wound healing after surgery and trauma^24^, but also abnormally during carcinogenesis and metastasis. Angiogenesis is a multistep process: beginning with an increase in permeability; proliferation by growth factors; new capillary sprout elongation^21,23,25^; proteolysis of the basement membrane; capillary channel formation; and finally, tube stabilization. ROS is a key mediator of microvascular hyperpermeability and endothelial barrier dysfunctions in BBB^8,26–28^. It has been shown that H_2_O_2_ increases endothelial permeability through protein kinase c signaling pathway and caspase-3 activation^8,29–31^. Uncontrolled ROS formation trigged by secondary injury induces endless pool of ROS leading to massive neuronal death. Apoptosis is important for regulating the normal development and removal of damaged cells and maintain a stable environment with in the cells. H_2_O_2_ induced apoptosis may be initiated by stimulating Ca^2+^ dependent endonuclease activity^7^. H_2_O_2_ can induce apoptosis in a concentration dependent manner in cerebral vascular smooth muscle cells^32^ and shown to induce apoptosis in vascular endothelial cells at concentrations greater than 125 µM^15,33^.

Although, reactive oxygen species are considered important in the physiologic regulation and pathophysiologic dysregulation of angiogenesis, hyperpermeability, and apoptosis, their relationship or role in the BBB has not been well-studied. It is important to understand how H_2_O_2_ affects the BBB differentially to help therapeutic drug development in trauma, ischemia and in a variety of diseases, as antioxidants and ROS scavengers are widely tested as drug targets. Therefore, the purpose of this study was to test the effect of varying concentrations of H_2_O_2_ on angiogenesis, barrier functions/permeability and cell viability/apoptosis to evaluate how it affects them differentially in BBB endothelial cells.

## Results

### Hydrogen Peroxide Induces Concentration-dependent Change in BBB Endothelial Cells

**Figure 1:**
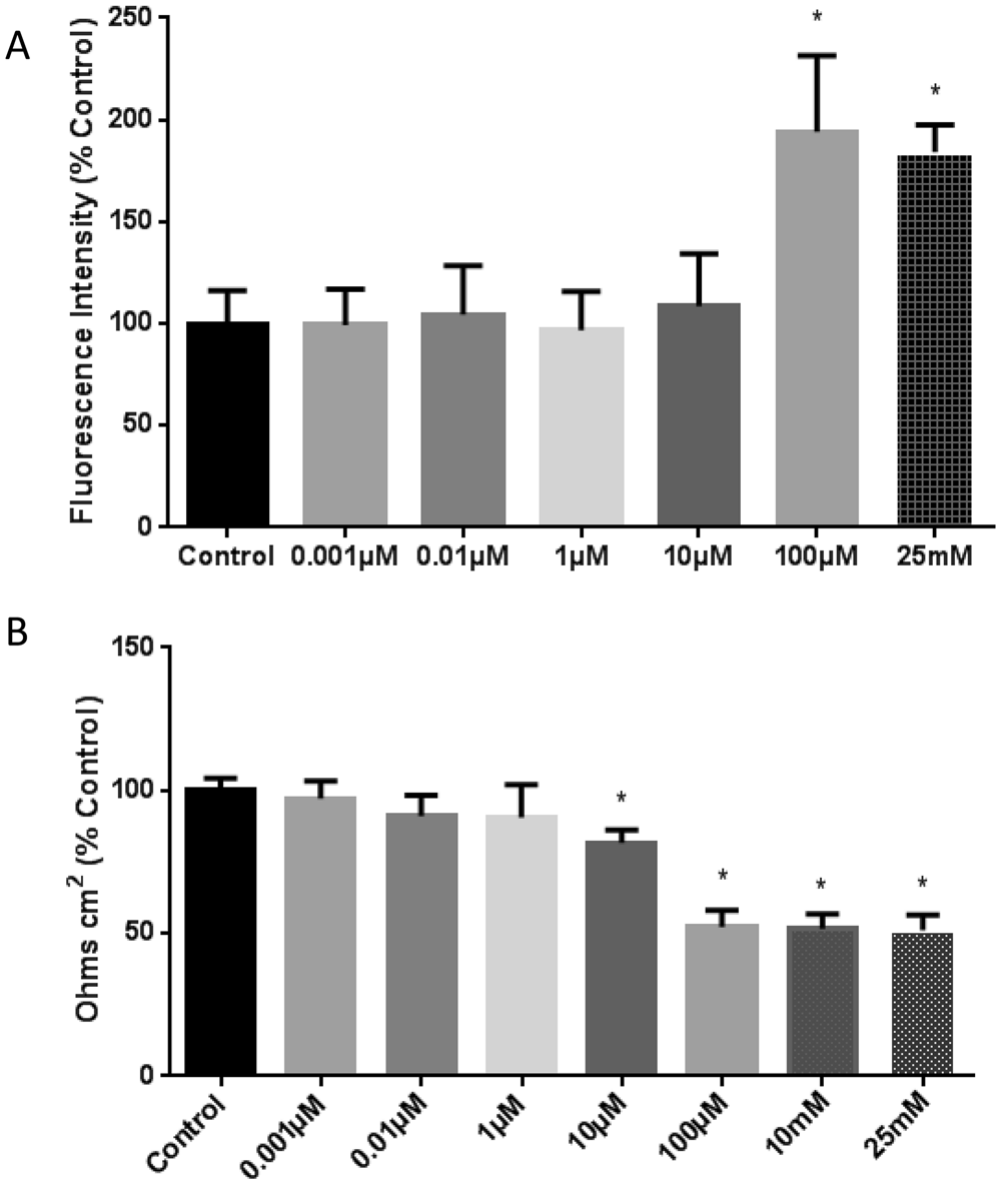
Monolayer Permeability assay demonstrating an increase in FITC-dextran fluorescence intensity as the concentration of H_2_O_2_ increases indicating barrier dysfunctions and hyperpermeability (**A**). Monolayer permeability is expressed as a percentage of the change in FITC-dextran-10 fluorescence intensity. ‘*’ indicates statistical significance (p < 0.05; n = 5); ‘*’ indicates significant change compared to control group (untreated group). Trans-endothelial electrical resistance measured following exposure to H_2_O_2_ showing a significant decrease in resistance as the concentration increases; ‘*’ indicates significant decrease in resistance in the cells treated with higher concentrations of H_2_O_2_ (10 µM to 25 mM) compared to control (p < 0.05; n = 6) (**B**).

Hydrogen peroxide at 100 µM and 25 mM (2 hours) induced monolayer permeability significantly indicated by increased fluorescence intensity in media collected from the lower reservoir of the Transwell plate (p < 0.05; Fig. 1A). H_2_O_2_ at concentrations 0.001 µM to 10 µM had no significant effect on permeability compared to the untreated control group. This shows that concentrations 100 µM and above are critical in promoting oxidative stress induced loss of BBB endothelial integrity resulting in hypermeability. This study was further confirmed by trans-endothelial electrical resistance (TEER) measurement. The results from TEER measurement indicated that the lower concentrations of H_2_O_2_ (0.001 to 1 µM) showed no significant change in TEER compared to the untreated control group, whereas a significantly low resistance was observed (p < 0.05; n = 4; Fig. 1B) in cells treated with concentrations of H_2_O_2_ (10 µM) and above compared to control.

### Hydrogen Peroxide Disrupts BBB Endothelial Cell Tight Junctions

**Figure 2:**
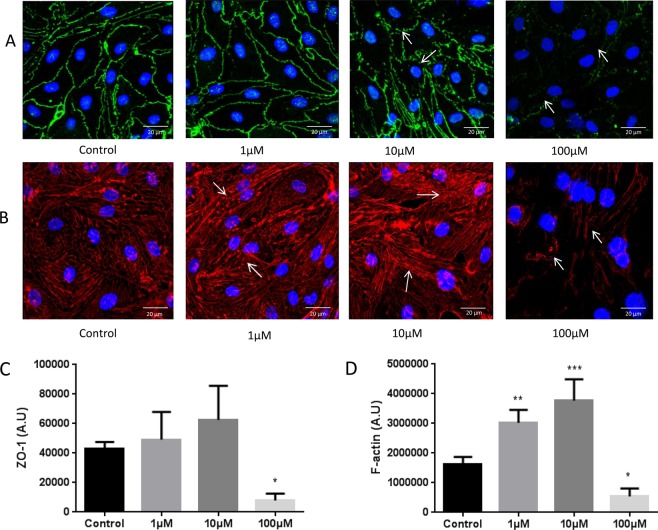
Immunofluorescence localization of tight junction protein ZO-1 and *f*-actin staining demonstrating disruption of the tight junctions/cytoskeletal disorganization following treatment with increasing concentration of H_2_O_2_ (for 2 hrs) in RBMECs. Treatment with 10 µM H_2_O_2_ led to clear breaks in the cell periphery/cell-cell junctions staining pattern (**A**, arrows) and a decrease in its localization compared to control group (**C**, asterisk). ‘*’ indicates statistical significance (p < 0.05; n = 4). The breakdown in the peripheral staining pattern/loss at the cell-cell junctions was more pronounced at the 100 µM treatment (**A**). Arrows indicate tight junction disruption. (**B**) Rhodamine phalloidin staining for *f*-actin demonstrating changes in cytoskeletal assembly evidenced by increased stress fiber formation following treatment with increasing concentration of H_2_O_2_ (for 2 hrs) in RBMECs. 1 µM and 10 µM H_2_O_2_ concentrations increased the formation of *f*-actin stress fibers. 100 µM showed disruption of the actin cytoskeletan (**D**). ‘*’ indicates statistical significance (p < 0.05; n = 4); control group. ‘**’ indicates significant change (p < 0.05) compared to control group. ‘***’ indicates significant change (p < 0.001) compared to control group (**B**). Arrows indicate actin stress fiber formation. Each experimental group consisted of 2 replicates. ‘Control group’ indicates an untreated group.

The immunofluorescence localization of tight junction protein ZO-1 demonstrated the disruption of tight junctions compared to control, whereas cells treated with 100 µM H_2_O_2_, for 2 hour, clearly indicated, disruption of cell-cell junctions and barrier damage. The disruption was observed, starting at 10 µM and was further obvious at 100 µM. Further quantitative analysis showed a significant decrease in ZO-1 localization at 100 µM concentration (p < 0.05; Fig. 2A,C) compared to all other groups (Control, 1 µM, 10 µM).

### Hydrogen Peroxide Disrupts BBB Endothelial Cell Cytoskeletal Organization

Rhodamine phalloidin labelling showed changes in cytoskeletal assembly in response to various treatments compared to normal cells. Cells treated with low concentrations of H_2_O_2_ (starting at 1 µM and 10 µM for 2 hour), increased the formation of f-actin stress fibers compared to the control (Fig. 2B,D; p < 0.05). Treatment with H_2_O_2_ (100 µM; 2 hour) showed disruption of actin cytoskeletan and on quantitative evaluation, a significant decrease in fluorescence intensity compared to the control (Fig. 2B,D; p < 0.05).

### Increasing H_2_O_2_ Concentrations Decrease Viability of BBB Endothelial cells

**Figure 3:**
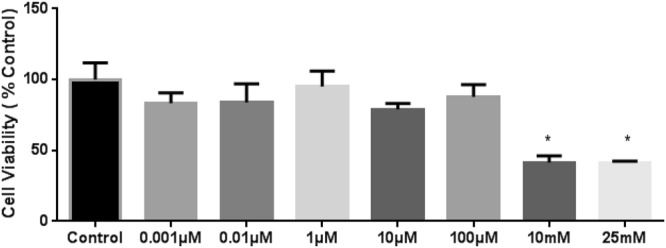
Hydrogen peroxide treatment decreases cell viability at higher concentrations. Cell viability assay was performed to measure the number of viable (living) cells remaining after H_2_O_2_ treatment (2 hours). The cells treated with higher concentrations of H_2_O_2_ showed a significant decrease in cell viability. ‘*’ indicates statistically significant difference compared to control (*p* < 0.05; n = 5).

Treatment of H_2_O_2_ at concentrations of 10 mM and 25 mM for 2 hours resulted in significant decrease in cell viability (p < 0.05; Fig. 3) whereas concentrations of 0.001 µM, 0.01 µM, 1 µM, 10 µM 100 µM showed no significant effect on cell viability (Fig. 3).

### Increasing H_2_O_2_ Concentrations Cause Apoptosis of BBB Endothelial cells

**Figure 4:**
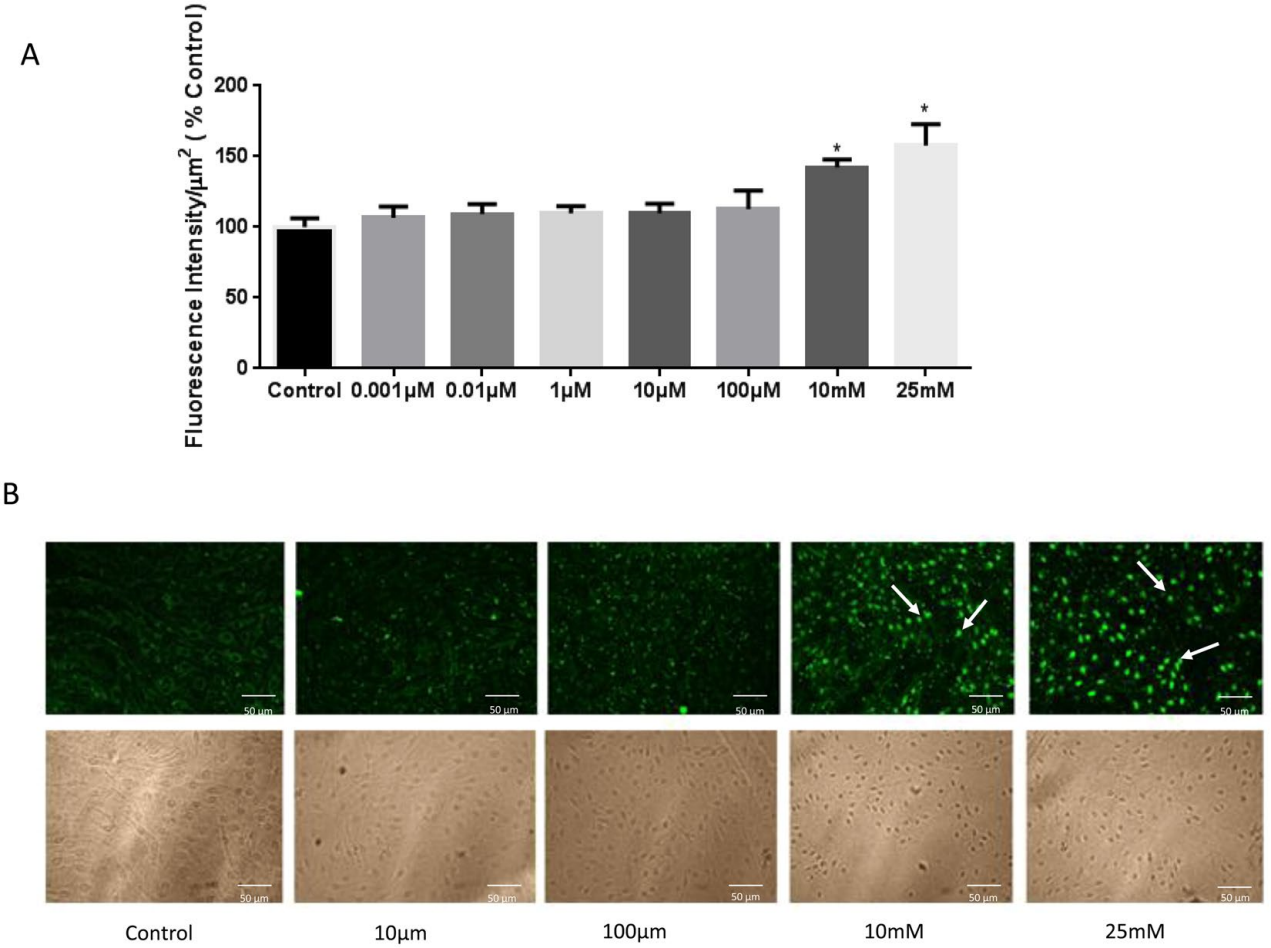
Hydrogen peroxide treatment increases apoptosis at higher concentrations evaluated by caspase-3/7 apoptosis assay (**A**). H_2_O_2_ at 10 mM and 25 mM induces apoptosis ‘*’ indicates statistically significant increase compared to control group (p < 0.05; n = 4). Hydrogen peroxide treatment increases apoptosis at higher concentrations when studied using TUNEL assay (**B**; n = 5). H_2_O_2_ at 10 mM and 25 mM induces apoptosis (some of the apoptotic cells are indicated by white arrows).

Apoptosis was evaluated using two different assays. RBMECs exposed to H_2_O_2_ at 10 mM and 25 mM resulted in apoptosis compared to untreated control and other lower concentrations tested (p < 0.05; Fig. 4A). The cells exposed to the lower concentration H_2_O_2_ (0.001 to 100 µM) showed no evidence of apoptosis when evaluated using an apoptosis assay (Fig. 4A). Images were taken and fluorescence intensity was measured using image-J. The apoptosis was further confirmed using TUNEL Alexa Fluor Imaging Assay. The analysis of the data, showed that cells treated with the higher concentrations of H_2_O_2_ (10 mM and 25 mM) induced apoptosis (p < 0.05; Fig. 4B) whereas low concentrations (10 µM and 100 µM) showed no evidence of apoptosis.

### Hydrogen Peroxide Induces Angiogenesis in Blood-Brain Barrier Endothelial cells

**Figure 5:**
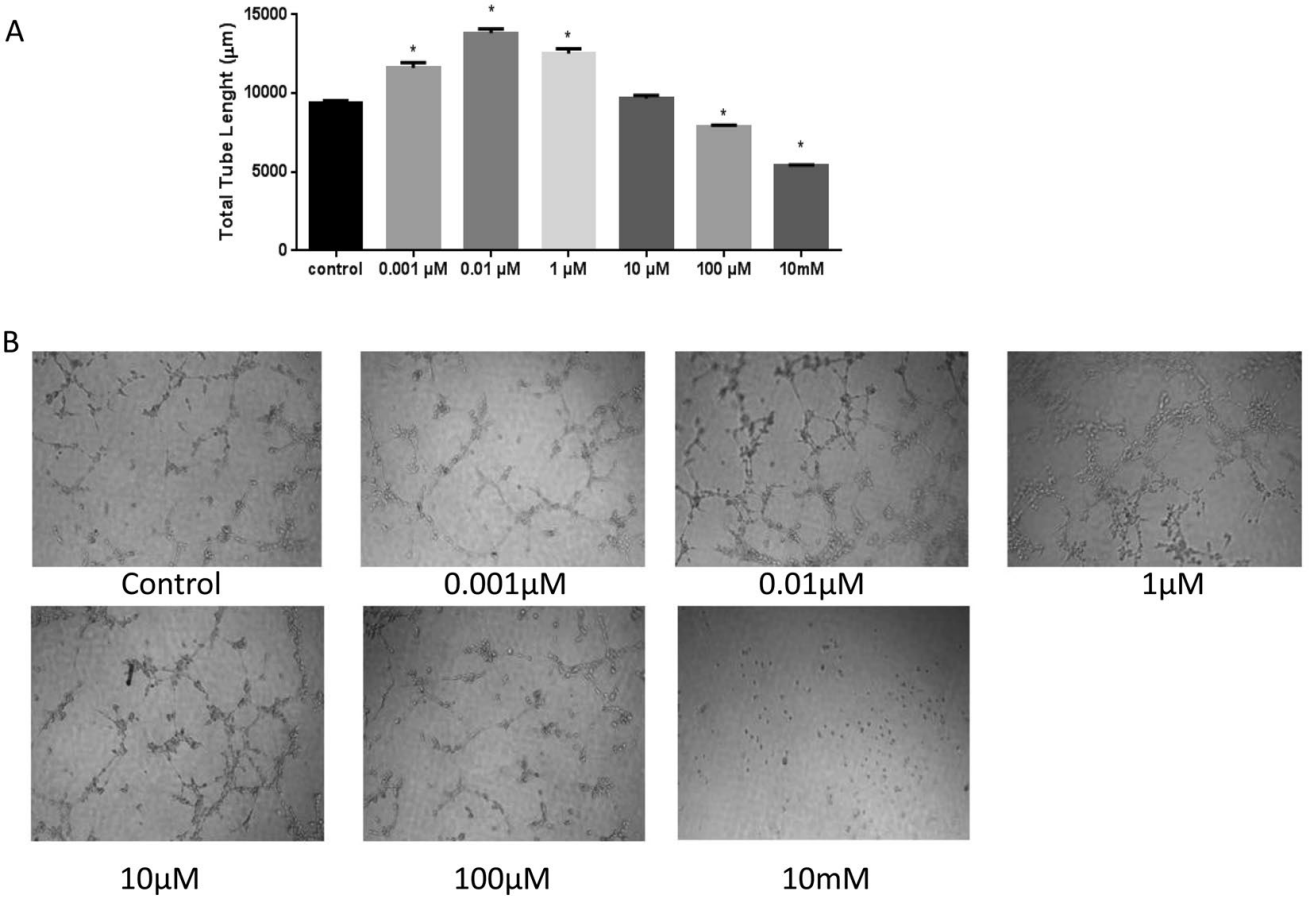
Effect of increasing concentrations of H_2_O_2_ on angiogenesis. The cells were treated with increasing concentrations of H_2_O_2_ (2 hrs) after seeding them in Matrigel and observed after 4 hrs. The cells treated with lower concentrations (0.001 µM, 0.01 µM, 1 µM) of H_2_O_2_ showed a significantly increase in angiogenesis (**A**). ‘*’ indicates statistically significant difference compared to control/untreated group (*p* < 0.05; n = 3). The lower panel (B) shows original images.

The change in angiogenesis was visualized and further quantitatively measured using image-J. Treatment with the lower concentrations of H_2_O_2_ (0.001 to 1 µM) resulted in significant increase in total endothelial cell tube length compared to the control (p < 0.05; Fig. 5A,B).

### Effects of H_2_O_2_ on Tight Junction Protein Expression

**Figure 6:**
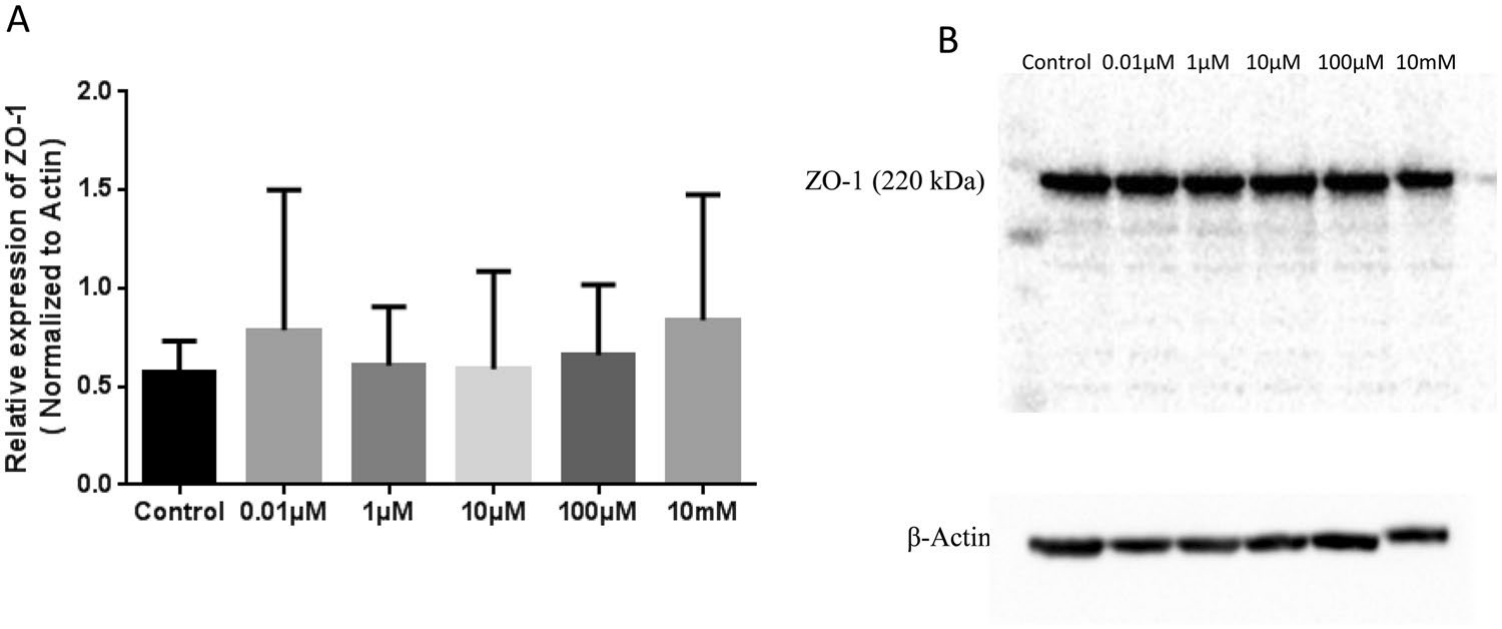
Immunoblot analysis of the tight junction protein ZO-1 demonstrating the effect of increasing concentrations of H_2_O_2_ on ZO-1 protein expression in RBMECs (**A** and **B**). Hydrogen peroxide treatment had no effect on ZO-1protein expression at any of the tested concentrations. The protein was normalized to the expression of β-actin (n = 5). The original images are given as supplementary figures (Supplementary Figs 3 and 4).

Immunoblot analysis showed the presence of tight junction protein ZO-1 (Fig. 6). Hydrogen peroxide treatment from 0.01 to 10 mM showed no significant change in ZO-1 protein expression. ZO-1 protein was normalized to the expression of β-actin for quantitative analysis (n = 5).

### Evaluation of the effects of H_2_O_2_ on endothelial cell senescence

H_2_O_2_ is known to induce pre-mature senescence in cells. This study was conducted to determine if the hyperpermeability observed (with 100 µM and above) was due to H_2_O_2_-induced pre-mature senescence in RBMECs. Senescent cells normally display increase of cell size and senescence-associated expression of β-galactosidase (SA-β-Gal) activity. The results show that H_2_O_2_ treatment didn’t induced pre-mature senescence compared to the control as there was no SA-β-Gal activity observed. The cells exposed to H_2_O_2_ (100 µM) for 2 hours and maintained for 72 hours showed evidence of senescence/SA-β-Gal activity (Supplementary Fig. 1).

### Visualization of intracellular H_2_O_2_

Intracellular H_2_O_2_ levels were visualized to confirm the presence of H_2_O_2_ during the treatment period in RBMECs. The results show that H_2_O_2_ is present intracellularly following treatment of H_2_O_2_ (Supplementary Fig. 2).

## Discussion

The purpose of the study was to evaluate the relationship between angiogenesis, permeability and cell death/apoptosis in BBB endothelial cells following H_2_O_2_ exposure to understand why H_2_O_2_ behave differentially in these conditions. The important observation of the study are: (1) low levels of H_2_O_2_ can cause angiogenesis (2) H_2_O_2_ plays an important role in promoting microvascular permeability by inducing tight junction disruption and cytoskeletal disorganization as its levels increases; (3) H_2_O_2_ when reaches a higher cellular level promotes cell death/apoptosis; (4) H_2_O_2_-mediated cellular changes are dependent on the tight junction organization evidenced by changes in Zonula Occludens-1 (ZO-1) and cytoskeletal assembly. All these effects are dependent on the changes in cellular levels of H_2_O_2._ Altogether these results demonstrate a tri-phasic role for H_2_O_2_ in regulating multiple cellular functions in brain microvascular endothelial cells, the primary components of the BBB and explain the basis of the various cellular manifestations subjected to changing levels of cellular H_2_O_2_ and possibly the oxidative stress status of these cells.

Although H_2_O_2_ has been previously shown to induce a wide variety of cellular functions such as angiogenesis, permeability and cell death, their relationship or underlying cellular mechanisms are not clearly known. Furthermore, the role played by H_2_O_2_ in regulating BBB-related functions remained unknown. The implications of our findings imply to a wide variety of disease conditions where oxidative stress and/or H_2_O_2_ play a significant role in its pathophysiology such as in traumatic and ischemic brain injury, multiple sclerosis, Alzheimer’s disease and Parkinson’s disease^24,34,35^. In addition to this, impairment of the BBB has been implicated in all the above conditions^8,13,17^.

Angiogenesis is an important function for growth, wound healing, and in the pathologic process of tumorigenesis^19,36^. Several studies have shown that H_2_O_2_ stimulates endothelial cell proliferation in different cell types but brain microvascular endothelial cells, the primary components of the BBB remained an unexplored area. Our study utilized isolated rat brain microvascular endothelial cells cultured on Matrigel, comparable to normal angiogenesis process in the brain. Endothelial tube formation is considered a critical step in *in vitro* angiogenesis. After the exposure to H_2_O_2_, a significant increase in tube formation was observed compared to the control group, demonstrated that low concentration of H_2_O_2_ can cause angiogenesis whereas the higher concentrations of H_2_O_2_ inhibit the tube formation.

Blood-brain barrier breakdown leads to microvascular hyperpermeability, the excessive leakage of proteins and fluid from the intravascular space to the insterstitium^10,13,17,37^. Clinically this is manifested as cerebral edema, increased intracranial pressure, and brain herniation^38–40^. Our results explain how H_2_O_2_ promotes microvascular permeability in the blood-brain barrier. Hydrogen peroxide at a concentration of 100 µM lead to increased permeability, decreased trans-endothelial electrical resistance (started observing from 10 µM) and also showed disruption of the tight junctions evidenced by ZO-1 localization, tight junction morphology and organization. However, none of the concentrations included in this study led to a change in overall ZO-1 protein levels. This could be due to the reason that higher H_2_O_2_ concentrations resulted in promoting the dislocation of ZO-1 from the tight junctions without affecting the protein expression/content. This was further supported by our observation that f-actin stress fibers were increased parallel to ZO-1 dislocation. This is likely secondary to the change in ZO-1 localization at the tight junctions, as it serves as a scaffolding protein between tight junction proteins and actin cytoskeletal assembly.

Although there are many adverse effects for H_2_O_2,_ its beneficial effect on brain endothelial cells adds to its therapeutic potential for many types of neurological pathologies^10^. The effect of H_2_O_2_ on cell death/apoptosis has been observed in a variety of cell types. Most of these studies looked at longer exposure times and found that H_2_O_2_ concentrations between 50 µM and 500 µM induced apoptosis and even higher concentrations led to unregulated cell death, necrosis^41–44^. Our study was conducted to determine the basis of its effect in a comparative manner and also in the context of the BBB testing it in rat brain microvascular endothelial cells. Apoptosis was evaluated by a caspase-3/7 assay and TUNEL assay. In this study H_2_O_2_ at a higher concentration of 10 mM, led to apoptosis and decreased cell viability. Although this suggest the adverse effects H_2_O_2_, this programmed cell death is important in wound healing and the prevention of tumorigenesis^7,45^. In our study, hydrogen peroxide at 100 µM induced hyperpermeability but did not decrease cell viability or increased apoptotic cell death. This suggests that permeability occurs independently of cell death. The increase in permeability could be attributed to changes in barrier integrity and compromised cytoskeletal organization. Although H_2_O_2_ at 25 mM decreased cell viability and 100 µM did not, their effect on permeability remained comparable. It is possible that permeability due to H_2_O_2_ at 100 µM reached its maximum and any further increase was not possible. This is evident from the data from the TEER study as well.

In order to determine if H_2_O_2_-induced-hyperpermeability is related to potential pre-mature senescence of endothelial cells, we conducted a senescence assay. Our results show that the concentrations of H_2_O_2_ that induce hyperpermeability doesn’t induce pre-mature senescence in endothelial cells. The ability of cells to remove extracellular H_2_O_2_ may vary between different cell types/cell lines. We conducted an H_2_O_2_ visualization assay to demonstrate intracellular presence of H_2_O_2_ following exposure to H_2_O_2_. Our results show the presence of detectable H_2_O_2_ intracellularly during the treatment period.

Our results clearly demonstrate a concentration dependent effect of H_2_O_2_ in regulating three significant cellular effects such as permeability regulation, angiogenesis and cell viability. As with any experiment, there are limitations of this study. This is an *in vitro* study conducted in rat brain microvascular endothelial cells and with increasing complexity with the small animal or human models, it is not known how H_2_O_2_ differentially regulates these functions. Rat brain microvascular endothelial cells are ideal tools for evaluating blood-brain barrier-related studies. The clinical implications of this study are unclear at this point. In a clinical context, considering the extensive use of non-prescription antioxidant medications and identification of newer antioxidant therapeutic drugs for various ailments, this study merits immense significance. In the meantime, H_2_O_2_ being a cellular signaling molecule, this study provides a clear basis for its multiple but differential effects in the blood-brain barrier. The tight junctions of endothelial cells are the most important barrier component of the BBB; however, this is a complex system made up of pericytes, astrocytes, and neurons and the monolayer permeability assay is an imperfect simulation. In conclusion, H_2_O_2_ has a tri-phasic role in rat brain microvascular endothelial cells and the understanding of these physiologic and pathologic effects will aid in the study of the BBB and neurovascular pathologies and in conditions such as traumatic brain injury and ischemic stroke.

## Materials and Methods

### Chemicals and Reagents

Rat Brain microvascular endothelial cells and Media were purchased from Cell Applications, Inc. (San Diego, CA). Transwell insert plates (24 well) were obtained from Corning, USA. Fibronectin (5%) from bovine plasma, chamber slides and FITC-Dextran-10 were purchased from Sigma Aldrich (St. Louis, MO, USA). DMEM, MEM, ZO-1 primary antibody, rhodamine phalloidin, Cell Event Caspase-3/7 Green Detection Reagent, Pierce BCA protein assay kit and he Click-iT® TUNEL Alexa Fluor® 488 Imaging Assay Kit were purchased from ThermoFisher Scientific (Waltham, MA, USA). Angiogenesis assay kit was bought from EMD Millipore/Calbiochem (Billerica, MA, USA). Cell viability assay kit, Senescence assay Kit and intracellular H_2_O_2_ detection Kit were purchased from Bio vision (Milpitas, CA, USA).

### Endothelial Cell Culture

Primary cultures of rat brain microvascular endothelial cells obtained commercially, as described above, were grown on fibronectin coated cell culture dishes, using rat brain endothelial media in optimal cell culture condition (95% O_2_, 5% CO_2_ at 37 °C). Once the cells attain 60–80% confluency, cells were transferred to Transwell inserts, chamber slides, 96 well plate or regular dishes as appropriate for experimental purposes. For all the experiments, cells of 6–9 passages were utilized.

### Monolayer Permeability Assay

RBMECs were grown on Transwell membranes coated with fibronectin until it formed a monolayer. It usually takes 4–5 days to form a monolayer. They were exposed to different concentrations of H_2_O_2_ (0.001 µM, 0.01 µM, 1 µM, 10 µM, 100 µM, 25 mM) for 2 hours. After treatment the FITC-dextran (10 kDa) was applied to the upper chamber incubate for 30 minutes. Samples were collected from the lower chamber. The Fluorescence intensity of the samples were measured using spectrophotometer. Each experimental group consisted of five replicates

### Trans Endothelial Electrical Resistance (TEER) Measurement

Transendothelial electrical resistance was measured using EVOM2. Rat brain microvascular endothelial cells were grown on Transwell membranes for 4 days and then exposed to different concentrations of H_2_O_2_ (0.001 µM, 0.01 µM, 1 µM, 10 µM, 100 µM, 10 mM and 25 mM) for 2 hours as described above. After the treatment with H_2_O_2_ TEER was measured. The changes in resistance was calculated as percentage (%) control.

### Immunofluorescence localization and cytoskeletal labeling

Rat brain microvascular endothelial cells were grown on 8-well chamber slides coated with fibronectin for 24 hours followed by treatment with different concentrations of H_2_O_2_ (1 µM, 10 µM, 100 µM, 2 hours). The cells were washed with phosphate buffered saline (PBS) and fixed with 4% paraformaldehyde in PBS for 15 minutes. After fixation the cells were permeabilized using 0.25% Triton-X 100 in PBS followed by blocking with 2% BSA in PBS for one hour at room temperature. For immunofluorescence study, the cells were incubated with primary antibody for ZO-1 for overnight, and the cells were incubated with secondary antibody for one hour in room temperature followed by washing in PBS and a mounting medium containing DAPI. For f-actin labelling, cells were exposed to rhodamine phalloidin for 20 minutes followed by washing and mounting as describe above. Cells were observed under a Confocal Microscope (60X water immersion objective).

### Cell Viability Assay

Rat brain microvascular endothelial cells were grown on black 96 well plates for 24 hours. Once the cells formed a monolayer, they were washed in PBS and exposed to phenol red-free medium for 1 hour. Cells were then treated with different concentrations of H_2_O_2_ (0.001 µM, 0.01 µM, 1 µM, 10 µM, 100 µM, 10 mM and 25 mM; 2 hours). After the treatments, Calcein buffer solution was applied to the cells and incubated at 37 °C for 30 minutes and a fluorescence intensity was measured. Each experimental group consisted of 5 replicates. Fluorescence intensity was expressed as percentage of the control.

### Apoptosis Assay

Cell Event Caspase-3/7 Green Detection Reagent was used to study cellular apoptosis with exposure to different concentrations of H_2_O_2_ (0.001 µM, 0.01 µM, 1 µM, 10 µM, 100 µM, 10 mM, 25 mM; 2 hours). This reagent is a four amino acid peptide, DEVD, conjugated to a nucleic acid binding dye with absorption/emission maxima of ~502/530 nm. The DEVD peptide sequence is a cleavage site for caspase-3/7 and the conjugate dye is non-fluorescent until cleaved from the peptide and bound to DNA. Therefore, the reagent is non-fluorescent until activation of caspase-3/7 in apoptotic cells leads to cleavage of the DEVD peptide. This allows the dye to bind to DNA and produce bright, fluorogenic nuclei. RBMECs were grown on chamber slides as described above and divided into control (untreated) and different concentrations of H_2_O_2_ (0.001 µM, 0.01 µM, 1 µM, 10 µM, 100 µM, 10 mM, 25 mM; 2 hours). Following this, Cell Event Caspase-3/7 Apoptosis Detection Reagent was added and cells were incubated at 37 °C for 30 minutes. Cells were then visualized and scanned at a single optical plane with Olympus Confocal Microscope. Each experimental group consisted of 4 replicates.

### TUNEL Alexa Fluor Imaging Assay

Tunnel Assay was conducted to measure apoptotic cells. RBMECs were grown on black 96-well plates with clear bottom. The cells were exposed to different concentrations of H_2_O_2_ (10 µM, 100 µM, 10 mM, 25 mM) for 2 hours. Cells were washed in PBS and then fixed with 4% para formaldehyde in PBS and permeabilized with 0.25% Triton X-100. TdT (terminal deoxynucleotidyl transferase) reaction mixture was added to the cells followed by Click-iT® Reaction Cocktails. Cells were washed in 3% BSA in PBS and observed under a Confocal Microscope.

### Angiogenesis Assay

Angiogenesis assays were performed by using RBMEC grown in RBMEC culture media. The cells were harvested and the cell suspensions containing 150,000 cells in 150 µl of RBMEC media and were seeded in triplicates on Matrigel coated glass bottom 96 well plates. Then they were treated with different concentrations of H_2_O_2_ for 2 hrs. After seeding the cells, the plates were incubated for 4 hrs at 37 °C and 5% CO_2_. The cells were observed after 4 hr. Tube formation was visualized using a phase contrast inverted (confocal) microscope 20X magnification. Quantification of Tube Network was done using Image J with the Angiogenesis Analyzer plugin16. The results are expressed as mean ± SD.

### Western blot Analysis

RBMECs were grown on 100 mm dishes. After confluency of 80%, the cells were divided into untreated (control) and treated group. The cells were treated with different concentration of H_2_O_2_ for 2 hrs at 37 °C. Standard western blot was performed to determine the expression of ZO-1 in RBMEC. The samples were incubated with the primary mouse monoclonal anti ZO-1 antibody (1:250 dilutions) overnight at 4 °C and then incubated with the goat anti-mouse IgG-HRP conjugated secondary antibody. The each experiment was repeated five times. Equal amount of protein sample loading was verified by assessing β-actin expression.

### Senescence Detection Assay

Hydrogen peroxide is known to induce pre-mature senescence in cells. In order to determine, if H_2_O_2-_induced hyperpermeability was independent of pre-mature senescence of endothelial cells, cellular senescence was evaluated using a senescence detection assay kit, Senescent cells normally display increase of cell size and senescence-associated expression of β-galactosidase (SA-β-Gal) activity. The cells were grown on 12-well plates and exposed to different concentrations of H_2_O_2_ (10 µM, 100 µM and 10 mM; 2 hours) at 37 °C. The cells were washed in PBS, and fixed with a fixative solution for 15 minutes. After that the cells were washed twice in PBS, the staining solution was added to the cells and incubated overnight at 37 °C. The cells were observed under a microscope for bright field imaging for increased cell size and increased SA-β-Gal activity evident by a blue staining. As a positive control, the cells were treated with H_2_O_2_ for 2 hours, replaced with fresh media and maintained for 72 hours prior to SA-β-Gal staining. Long-duration H_2_O_2_ exposure is known to induce pre-mature senescence in endothelial cells.

### Visualization of intracellular H_2_O_2_

This assay was performed to confirm the presence of H_2_O_2_ intracellularly following exposure to H_2_O_2_. Intracellular hydrogen peroxide detection kit uses a dye that reacts with intracellular hydrogen peroxide to produce an orange color and fluorescence, which is proportional to the concentration of intracellular hydrogen peroxide. RBMECs (2–3 × 10^4^ cells/well) were grown on a 96-well plate. Cells were incubated overnight in 37 °C incubator containing 5% CO_2_. Next day, cells were treated with different concentration H_2_O_2_ (0.001 µM, 0.01 µM, 1 µM, 10 µM, 100 µM, 10 mM, 25 mM) or control for 2 hour in the presence of the Dye. Cells were examined using a fluorescence microscope (Excitation/Emission = 543 nm/545–750 nm). Images were acquired from each well.

### Statistical Analysis

The data from all experiments were analyzed and expressed as the mean +/− SEM (%).One-way analysis of variance (ANOVA) followed by Bonferroni test was performed to identify the groups that are statistically different (P < 0.05).

## Electronic supplementary material

Supplementary Information
