# Supplementary material for: The Tri-phasic Role of Hydrogen Peroxide in Blood-Brain Barrier Endothelial cells
Source: Sci Rep. 2019 Jan 15;9:133. doi: 10.1038/s41598-018-36769-3 (PMC6333800; doi:10.1038/s41598-018-36769-3)

The Tri-phasic Role of Hydrogen Peroxide in Blood-Brain Barrier Endothelial cells

Chinchusha Anasooya Shaji, Bobby D. Robinson, Antonia Yeager, Madhava R. Beeram, Matthew L. Davis, Claire L. Isbell, Jason H. Huang, Binu Tharakan

Supplementary Figure-1. Hydrogen peroxide-induced monolayer hyperpermeability is not due to pre-mature senescence of endothelial cells. A. H_2O_2 treatment at 10µM to 10mM for 2 hours did not induce senescence associated SA-β-Gal activity/blue staining (n=4). B. H_2O_2 treatment at 100µM for 72 hours (positive control) induced senescence evidenced by SA-β-Gal activity/blue staining (n=4).

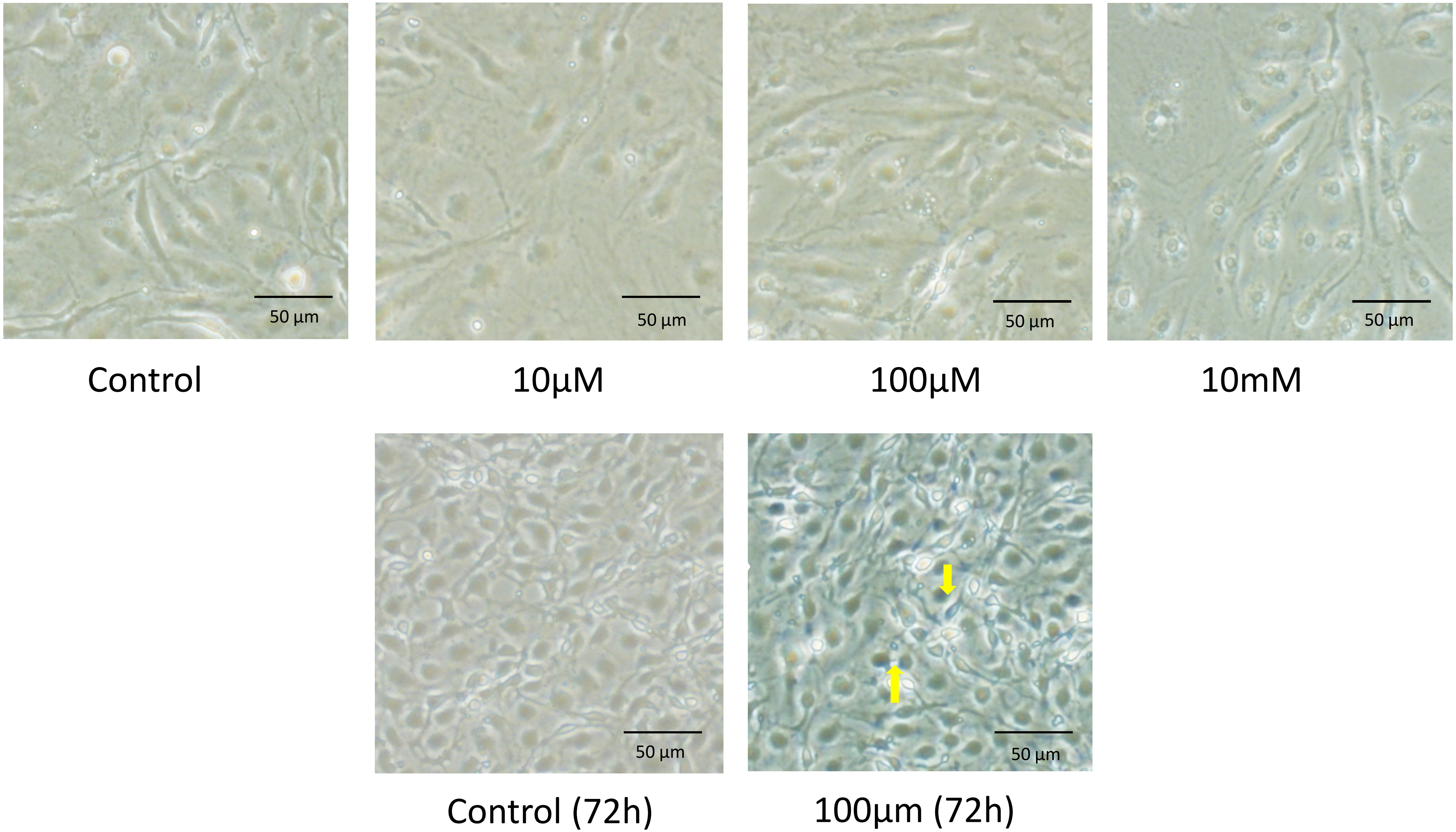
50 µm
50 µm
50 µm
50 µm
Control
10µM
100µM
10mM
50 µm
50 µm
Control (72h)
100µm (72h)

The Tri-phasic Role of Hydrogen Peroxide in Blood-Brain Barrier Endothelial cells

Chinchusha Anasooya Shaji, Bobby D. Robinson, Antonia Yeager, Madhava R. Beeram, Matthew L. Davis, Claire L. Isbell, Jason H. Huang, Binu Tharakan

Supplementary Figure-2. Hydrogen peroxide visualization assay demonstrating the presence of intracellular H_2O_2 following exposure to increasing concentrations of H_2O_2 (n=4)

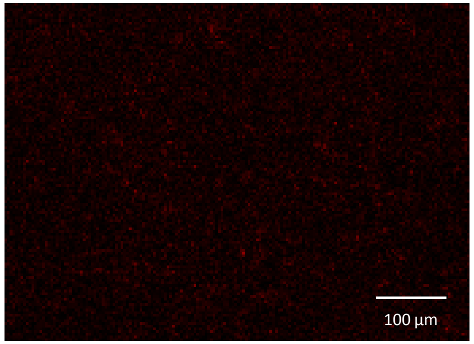

0 µM

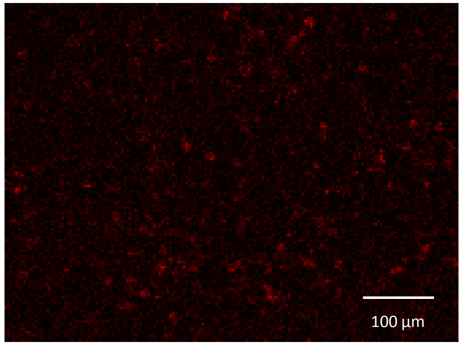

0.001 µM

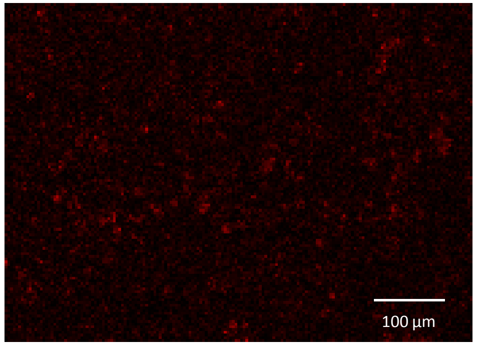

0.01 µM

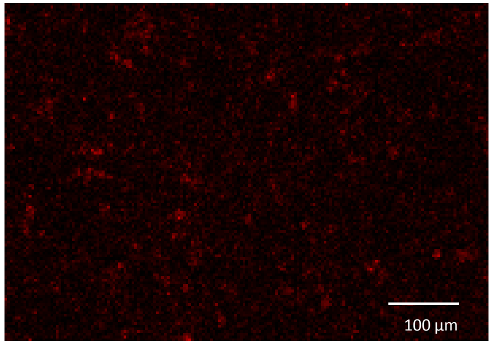

1 µM

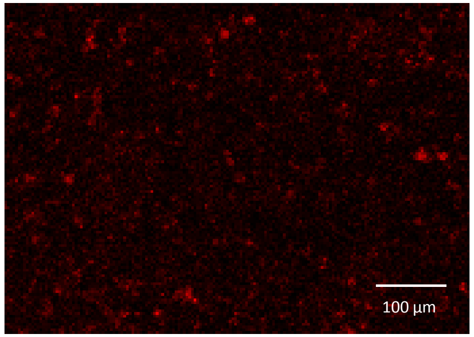

10 µM

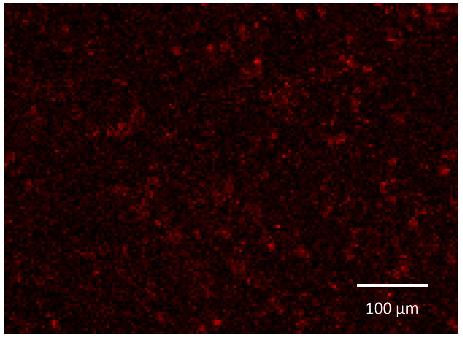

100 µM

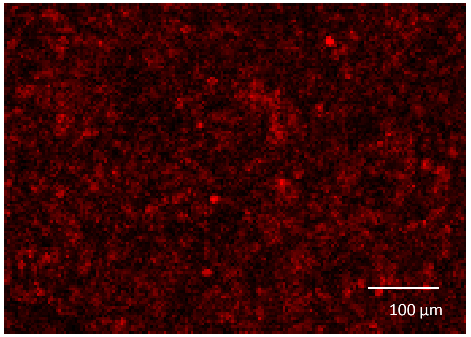

10 mM

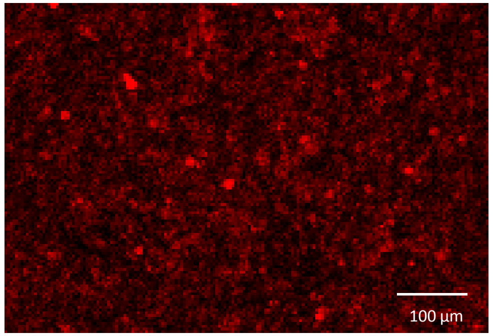

25 mM

The Tri-phasic Role of Hydrogen Peroxide in Blood-Brain Barrier Endothelial cells

Chinchusha Anasooya Shaji, Bobby D. Robinson, Antonia Yeager, Madhava R. Beeram, Matthew L. Davis, Claire L. Isbell, Jason H. Huang, Binu Tharakan

Supplementary Figure-3. Original immunoblot image of the data shown in figure 6 demonstrating the effect of increasing concentrations of H_2O_2 on ZO-1 protein expression in RBMECs. Hydrogen peroxide treatment had no effect on ZO-1protein expression at any of the tested concentrations.

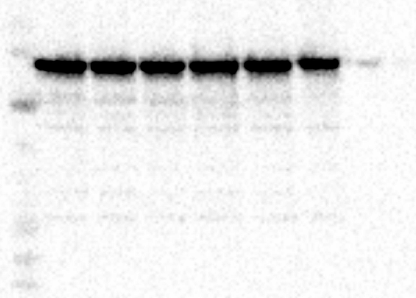

The Tri-phasic Role of Hydrogen Peroxide in Blood-Brain Barrier Endothelial cells

Chinchusha Anasooya Shaji, Bobby D. Robinson, Antonia Yeager, Madhava R. Beeram, Matthew L. Davis, Claire L. Isbell, Jason H. Huang, Binu Tharakan

Supplementary Figure-4. Original immunoblot image of the data shown in figure 6 demonstrating comparable levels of β-actin (internal control).

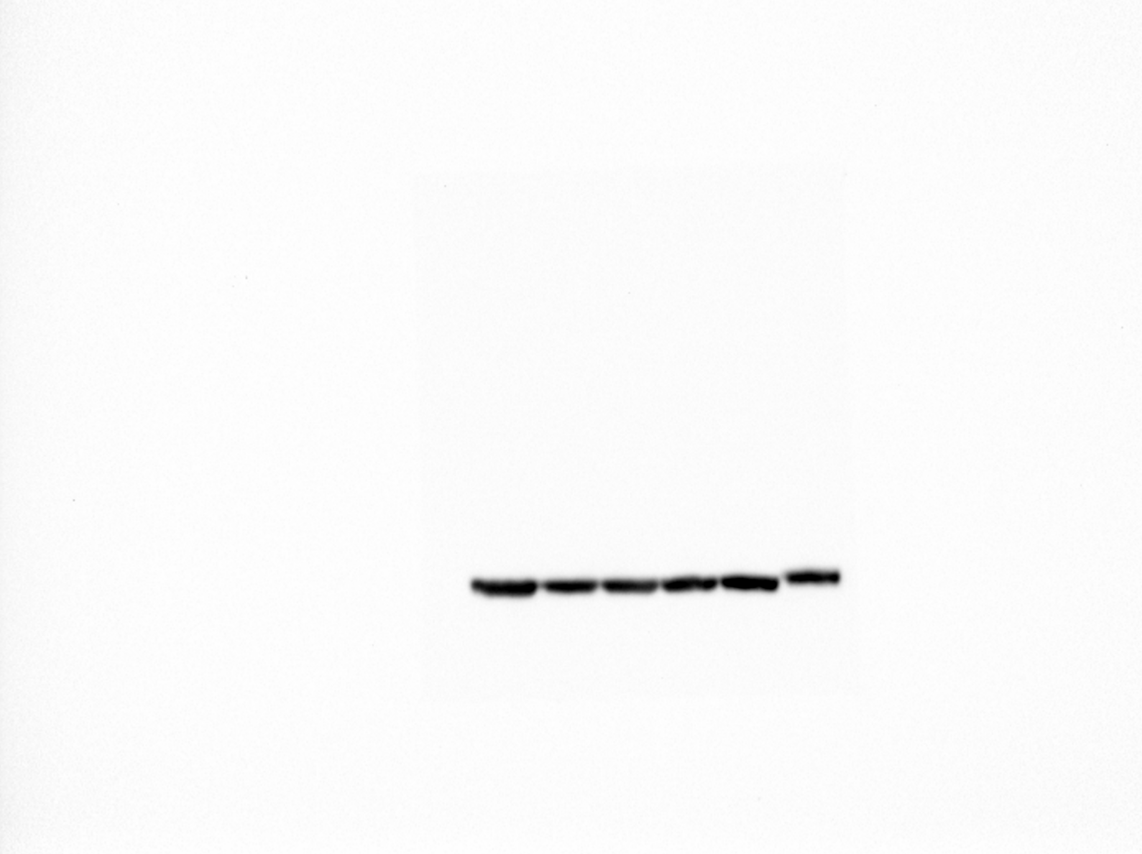

Supplement: Supplementary file 1 — Supplementary Information [file 41598_2018_36769_MOESM1_ESM.pdf]
